# Loss of HCN2 in Dorsal Hippocampus of Young Adult Mice Induces Specific Apoptosis of the CA1 Pyramidal Neuron Layer

**DOI:** 10.3390/ijms22136699

**Published:** 2021-06-22

**Authors:** Matthias Deutsch, Carina Stegmayr, Sabine Balfanz, Arnd Baumann

**Affiliations:** 1 Research Center Jülich, Institute of Biological Information Processing, IBI-1, 52428 Jülich, Germany; mdeutsch@UCSD.edu (M.D.); s.balfanz@fz-juelich.de (S.B.); 2 Department of Biology, University of California, San Diego, La Jolla, CA 92083, USA; 3 Research Center Jülich, Institute of Neuroscience and Medicine, INM-4, 52428 Jülich, Germany; c.stegmayr@fz-juelich.de

**Keywords:** Adeno-associated virus (AAV), hyperpolarization-activated and cyclic nucleotide-gated ion channel (HCN-channel), immunocytochemistry, knock-down, pacemaker channel, patch-clamp, signaling, transcript quantification, viral transduction, behavioral testing

## Abstract

Neurons inevitably rely on a proper repertoire and distribution of membrane-bound ion-conducting channels. Among these proteins, the family of hyperpolarization-activated and cyclic nucleotide-gated (HCN) channels possesses unique properties giving rise to the corresponding I_h_-current that contributes to various aspects of neural signaling. In mammals, four genes (*hcn1-4*) encode subunits of HCN channels. These subunits can assemble as hetero- or homotetrameric ion-conducting channels. In order to elaborate on the specific role of the HCN2 subunit in shaping electrical properties of neurons, we applied an Adeno-associated virus (AAV)-mediated, RNAi-based knock-down strategy of *hcn2* gene expression both in vitro and in vivo. Electrophysiological measurements showed that HCN2 subunit knock-down resulted in specific yet anticipated changes in I_h_-current properties in primary hippocampal neurons and, in addition, corroborated that the HCN2 subunit participates in postsynaptic signal integration. To further address the role of the HCN2 subunit in vivo, we injected recombinant (r)AAVs into the dorsal hippocampus of young adult male mice. Behavioral and biochemical analyses were conducted to assess the contribution of HCN2-containing channels in shaping hippocampal network properties. Surprisingly, knock-down of *hcn2* expression resulted in a severe degeneration of the CA1 pyramidal cell layer, which did not occur in mice injected with control rAAV constructs. This finding might pinpoint to a vital and yet unknown contribution of HCN2 channels in establishing or maintaining the proper function of CA1 pyramidal neurons of the dorsal hippocampus.

## 1. Introduction

Hyperpolarization-activated and cyclic nucleotide-gated (HCN) channels are known to control important electrical properties of neurons [1]. Due to their unique activation and gating properties, these channels play crucial roles in generating rhythmic cellular activities and thereby participate, e.g., in cardiac pacemaking [2] as well as in modulating the sleep and wake rhythm [3]. In contrast to these well characterized properties, the contribution of HCN channels to other neural functions is still elusive.

In mammals, the family of HCN channel genes comprises four different isoforms (*hcn1-4*), which can assemble as homo- or hetero-tetrameric ion-conducting channels [4]. In contrast to other voltage-gated channels, inward movement of the voltage-sensor upon membrane hyperpolarization triggers opening of HCN channels, resulting in a depolarizing inward current. In addition to the voltage dependence of HCN channel gating, their open state probability and activation kinetics are further modulated by cyclic nucleotides [5].

Due to channel activation close to resting membrane potentials and their non-inactivating current properties, HCN channels exert a depolarizing effect on resting membrane potentials in many cell types [6]. Thus, HCN channels might serve to stabilize resting membrane properties against both hyperpolarizing and depolarizing inputs [1]. This function of I_h_-currents is thought to influence dendritic integration properties in hippocampal CA1 pyramidal neurons by modulating the kinetics of excitatory and inhibitory postsynaptic potentials [7]. Since the biophysical properties of HCN-channel isoforms are strikingly different, the individual subtypes might be expressed differentially and/or even complementary. This hypothesis was further strengthened by the finding that hetero-oligomerization of HCN subunits to hetero-tetrameric ion channels occurred in various tissues and also changed during development [8,9]. Because the various combinations of hetero-tetrameric channels result in a multitude of biophysical I_h_-current properties, neurons possess a versatile mechanism to generate specific I_h_-currents to fulfill their physiological requirements. The existence of a plethora of β-subunits, scaffolding proteins, and regulatory proteins, i.e., TRIP8b, Caveolin-3, or MiRP1, even increases the spectrum and may add further variability to their biophysical properties [10].

HCN-channel expression has been described in numerous cell types throughout the mammalian central nervous system (CNS) [11]. Especially in the hippocampus, a region known to play a critical role in learning and memory [12], HCN channels are highly expressed in developing and adult animals [13]. Notably, both electrophysiological and immunohistochemical studies revealed that I_h_-current and the HCN1 and HCN2 subunits are enriched in the distal dendrites of hippocampal pyramidal neurons [14,15,16,17]. Although at lower densities, HCN channels are also located on the soma of some pyramidal neurons [18]. Impairing the I_h_-current by pharmacological blockade or genetic ablation results in augmented dendritic excitability [14,15,18,19]. The changes in membrane properties ultimately affect synaptic potentials and thus also the dendritic integration properties of pyramidal neurons [20]. Moreover, HCN channel gene-expression undergoes developmental changes during aging [13]. Hence, native HCN channels are differently expressed, diversely regulated, and contribute to various cellular and network functions within the hippocampal formation. In order to elaborate on the contribution of individual HCN-channel subunits to neuronal and systemic functions, we utilized an Adeno-associated virus (AAV)-mediated gene knock-down strategy to specifically impair HCN2 subunit expression in both cultivated primary hippocampal neurons (PHNs) and in living mice. The main findings were that knock-down of *hcn2* expression in PHNs resulted in HCN channel isoform-specific changes of the I_h_-current. Surprisingly, stereotaxic injection of rAAVs causing HCN2 knock-down in the dorsal hippocampus led to a severe loss of neurons in the CA1 region. We attributed this effect to apoptotic rather than inflammatory processes. Furthermore, these apoptotic events were specific for the loss of HCN2 because they did not occur in animals that received rAAVs encoding control shRNAs. We assume that the loss of the HCN2 subunit in 8–12-week-old mice interferes with establishing and/or maintaining the proper neural network function and signaling in the hippocampal formation.

## 2. Results

### 2.1. RNAi-Mediated Knock-Down of the HCN2 Subunit in PHNs

**Figure 1:**
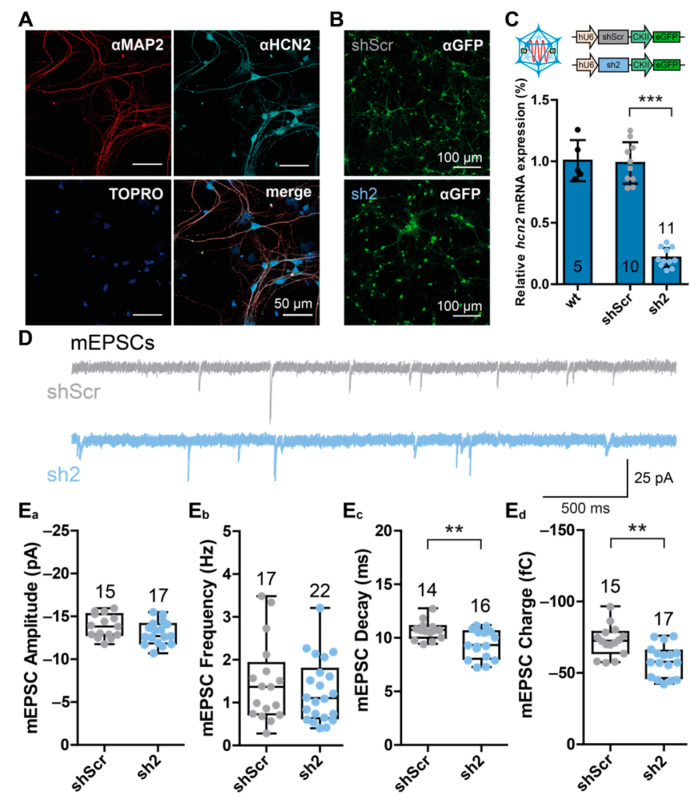
RNAi-mediated knock-down of *hcn2* transcripts altered dendritic integration properties of PHNs. (**A**) Representative immunofluorescence images showing the expression of the HCN channel isoform 2 (HCN2) in primary hippocampal neurons (PHNs). The neuron-specific MAP2 protein (red) and HCN2 proteins (cyan) were stained with specific anti (α)-MAP2 and α-HCN2 antibodies combined with fluorescently labeled secondary antibodies. Nuclei were labeled with TOPRO (blue). (**B**) Representative immunofluorescent images showing eGFP (green) expression in rAAV9-transduced PHNs either encoding control shRNA (shScr, upper image) or *hcn2*-specific shRNA (sh2, lower image). (**C**) qRT-PCR analysis of *hcn2* transcript levels of wildtype PHNs and PHNs treated with either shScr-expressing or sh2-expressing rAAVs. Transcript levels were normalized to *gapdh*, and values shown are normalized to wildtype *hcn2* transcript numbers. cDNA was prepared from indicated numbers of coverslips obtained from at least 3 different animals. Cartoons of the rAAV9 constructs are displayed above the images. (**D**) Representative current traces showing miniature excitatory postsynaptic currents (mEPSCs) in PHNs treated with shScr- (gray) or sh2-encoding (blue) rAAVs. (**E**) Influence of HCN2 knock-down on (**E_a_**) mEPSC amplitude; (**E_b_**) mEPSC frequency; (**E_c_**) mEPSC decay time, calculated by fitting the decay phase by a mono-exponential decay equation; and (**E_d_**) on mEPSC charge, calculated by the integral of the synaptic events. Data are depicted as boxplots. Numbers above boxplots indicate the number of neurons analyzed by patch-clamp electrophysiology. Statistical significance was assessed using the unpaired two-tailed Student’s *t*-test, ** *p* < 0.01; *** *p* < 0.001.

To assess the role of HCN2 subunit-containing channels in hippocampal neurons, we expressed specific shRNAs to knock down the HCN2 subunit by the inhibitory RNA (RNAi) mechanism. To quantify the knock-down efficacy and to assess the electrophysiological consequences of reduced HCN2 subunit expression, we utilized primary hippocampal neuron cultures (PHNs). These neurons express in particular the HCN2 subunit to a relatively high extent (Figure 1A) compared to HCN1 or HCN4 subunits [21]. As established in a previous study [21], recombinant Adeno-associated viruses (rAAVs) of serotype 9, which is considered transducing neurons with high specificity [22], were generated to deliver *hcn2*-specific constructs (sh2) to target cells. Successfully transduced neurons were identified by eGFP expression. A scrambled shRNA-encoding construct (shScr), which should not have any endogenous binding site, was used as an independent control (Figure 1B).

To verify the knock-down efficiency of the RNAi approach, we performed RT-qPCR analysis of either wildtype, shScr-treated, or sh2-treated PHNs. While no difference between untreated wildtype neurons and shScr-treated neurons (wt: 1.0 ± 0.167; shScr: 0.987 ± 0.168) was observed, there was a strong reduction of *hcn2* transcript numbers in sh2-treated neurons (sh2: 0.217 ± 0.078) (Figure 1C).

Because RNAi robustly reduced *hcn2* transcript levels and HCN2 is known to control integration of excitatory postsynaptic potentials (EPSPs) at dendrites of CA1 pyramidal neurons [19], we assessed the consequences of HCN2 channel knock-down on neuronal transmission. Miniature EPSCs (mEPSCs) were recorded in shScr-treated control neurons and sh2-treated neurons. In order to eliminate spontaneous activity emerging from the network, action potentials were blocked by application of 2 µM tetrodotoxin (TTX) in addition to the GABA_A_ receptor blocker bicuculline (25 µM). Neurons were clamped to −70 mV, and mEPSCs were recorded, induced by the probabilistic spontaneous release of neurotransmitters form the presynaptic side (Figure 1D).

The knock-down of HCN2 did not affect mEPSC amplitude (shScr: −13.84 ± 1.35 pA; sh2: −13.06 ± 1.35 pA) or mEPSC frequency (shScr: 1.47 ± 0.95 Hz; sh2: 1.28 ± 0.74 Hz) (Figure 1E_a_,E_b_). However, loss of HCN2 decreased mEPSC decay time (shScr: 10.68 ± 0.87 ms; sh2: 9.37 ± 1.35 ms) and consequently also mEPSC charge transfer (shScr: −72.28 ± 10.76 fC; sh2: −58.53 ± 11.0 fC) (Figure 1E_c_,E_d_), suggesting a postsynaptic role of HCN2 subunit-containing channels.

We also recorded spontaneous EPSCs (sEPSCs) to check for action potential-related influences on synaptic transmission. However, as action potential properties of control PHNs (shScr-treated) were not altered compared to sh2-treated PHNs (Figure S1A,B), loss of HCN2 affected sEPSCs similarly to mEPSCs (Figure S1C,D). Both sEPSC current amplitude and frequency were not affected, but sEPSC decay time and charge transfer were reduced in neurons lacking HCN2. These experiments indicate that a loss of HCN2 changes dendritic integration properties without affecting presynaptic mechanisms.

**Figure 2:**
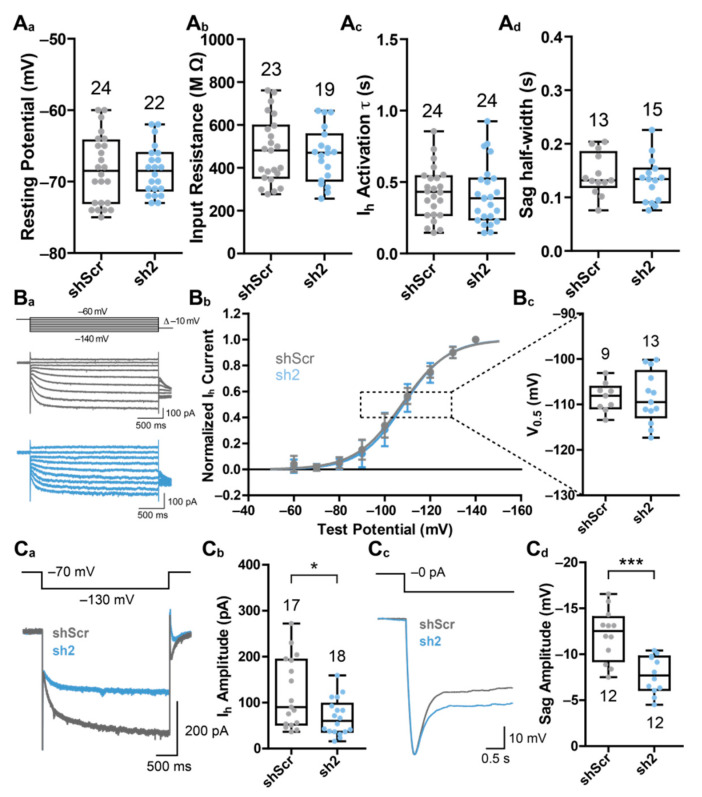
Electrophysiological effects of HCN2 knock-down in PHNs. Results of whole-cell patch-clamp recordings of rAAV9-transduced and eGFP-positive PHNs. (**A**) Effects of HCN2 knock-down on (**A_a_**) resting membrane potential, (**A_b_**) input resistance, (**A_c_**) I_h_-current activation time constant τ, and (**A_d_**) Sag-potential half-width. Resting membrane potentials were measured approximately 30 s after establishing the whole-cell configuration. Input resistances were measured with three 10 mV hyperpolarizing voltage pulses. Activation time constants were calculated by fitting the current response of a hyperpolarizing pulse (from −70 mV to −130 mV) to a mono-exponential function. Sag potentials were evoked by current pulses hyperpolarizing the membrane potential to −130 mV. (**B**) Effects of HCN2 knock-down on I_h_-current activation potentials. (**B_a_**) Representative voltage stimulation protocols and corresponding current traces of whole-cell patch-clamp recordings derived from PHNs expressing shScr (gray) or sh2 (blue). (**B_b_**) Current–voltage relationships of shScr- or sh2-expressing PHNs. Currents were calculated from the difference of the instantaneous current and the steady-state current. The continuous lines represent fitted Boltzmann functions of the recorded currents. (**B_c_**) Half-maximal activation voltages of shScr- or sh2-expressing PHNs calculated from the fitted Boltzmann functions of whole-cell currents. (**C**) Influence of HCN2 knock-down on I_h_-current amplitude and Sag-potential amplitude. (**C_a_**) Representative voltage stimulation protocol and corresponding current traces of PHNs expressing shScr (gray) or sh2 (blue). (**C_b_**) I_h_-currents were activated by a hyperpolarizing pulse (from −70 mV to −130 mV) and calculated from the difference of the instantaneous current and the steady-state current. (**C_c_**) Representative current stimulation protocol and corresponding voltage traces of PHNs expressing shScr (gray) or sh2 (blue). (**C_d_**) Sag potentials were evoked by current pulses hyperpolarizing the membrane potential to −130 mV. Results are depicted as boxplots. Numbers indicate the number of neurons analyzed by patch-clamp electrophysiology. Statistical significance was assessed using unpaired two-tailed Student’s *t*-test, * *p* < 0.05, *** *p* < 0.001.

To examine the electrical properties underlying the observed changes in synaptic transmission, we assessed the effects of HCN2 loss on PHN’s passive electrical as well as I_h_-current-specific properties. It was shown that the loss of HCN1 channels leads to changes in resting membrane potential [23]. However, we did not detect any differences in the resting membrane potentials of control neurons and neurons lacking HCN2 (shScr: −68.33 ± 4.82 mV; sh2: −68.45 ± 3.42 mV) (Figure 2A_a_). As an additional measure for the ability of neurons to conduct current at resting conditions, we measured input resistances. Again, we did not detect any differences in the input resistance of control neurons compared to neurons lacking HCN2 (shScr: 481.0 ± 153.9 MΩ; sh2: 458.9 ± 127.7 MΩ) (Figure 2A_b_). These results suggest that manipulation of *hcn2* gene expression neither influences the proportion of open HCN channels, nor the amount of current crossing the membrane at the resting membrane potential.

To analyze if a reduction of HCN2 expression might affect the kinetics of the HCN channel-mediated I_h_-current or its corresponding Sag-potential, we examined the time course of I_h_-current activation and the half-width of the Sag-potential. Neither the time course of current activation (shScr: 0.427 ± 0.182 s; sh2: 0.412 ± 0.211 s) (Figure 2A_c_) nor the Sag-potential half-width (shScr: 0.143 ± 0.039 s; sh2: 0.132 ± 0.042 s) (Figure 2A_d_) differed between control and HCN2 knock-down conditions, indicating that a loss of the HCN2 subunit does not influence I_h_-current kinetics or its corresponding Sag-potential kinetics.

Because loss of HCN1 induces changes in the I_h_-current–voltage relationship [21], we measured current–voltage relationships and half-maximal activation potentials (V_1/2_) of neurons lacking HCN2 and compared them to shScr-treated control neurons. Analogous to the I_h_-current activation kinetics and the Sag-potential half-width, we did not detect any changes in current–voltage relationships as well as half-maximal activation potentials (V_1/2_: shScr: −108.4 ± 3.2 mV; sh2: −108.5 ± 5.71 mV) (Figure 2B).

The robustness of the before described HCN-related parameters upon HCN2 knock-down was surprising, because *hcn2* transcript levels outnumber those of *hcn1* and especially those of *hcn4* [21]. Moreover, HCN2 channels possess the highest single channel conductance among the homomeric HCN channel subtypes [24]. Therefore, we examined if a reduction of *hcn2* expression affects the I_h_-current amplitude or its corresponding Sag-potential amplitude. Both the I_h_-current amplitude (shScr: 120.3 ± 76.1 pA; sh2: 67.8 ± 37.8 pA) (Figure 2C_a_,C_b_) and its corresponding Sag-potential amplitude (shScr: 12.09 ± 2.85 mV; sh2: 7.77 ± 2.04 mV) (Figure 2C_c_,C_d_) were strongly reduced in neurons lacking HCN2.

In summary, the reduction of HCN2 subunit expression did not influence passive membrane properties, I_h_-current activation kinetics, or I_h_-current–voltage relationships, but strongly decreased I_h_-current amplitudes, suggesting a role of HCN2 in controlling the number or the conductance of HCN channels present at the plasma membrane of PHNs.

Notably, we did not detect any differences in the calcium kinetics in the somata upon external stimulation of either shScr- (control) or sh2-treated PHNs (Figure S2). Thus, the effects of HCN2 knock-down on EPSP kinetics are most likely due to a perturbation of the expression level of HCN2-containing channels at the dendrites. In summary, the electrophysiological experiments suggested that the knock-down of HCN2 did not change action potential properties or calcium responses, but it reduced I_h_-current amplitudes and thereby contributed to alterations in dendritic integration properties of excitatory synaptic inputs in PHNs.

### 2.2. In Vivo HCN2 Knock-Down by Stereotaxic Intrahippocampal rAAV Injections

**Figure 3:**
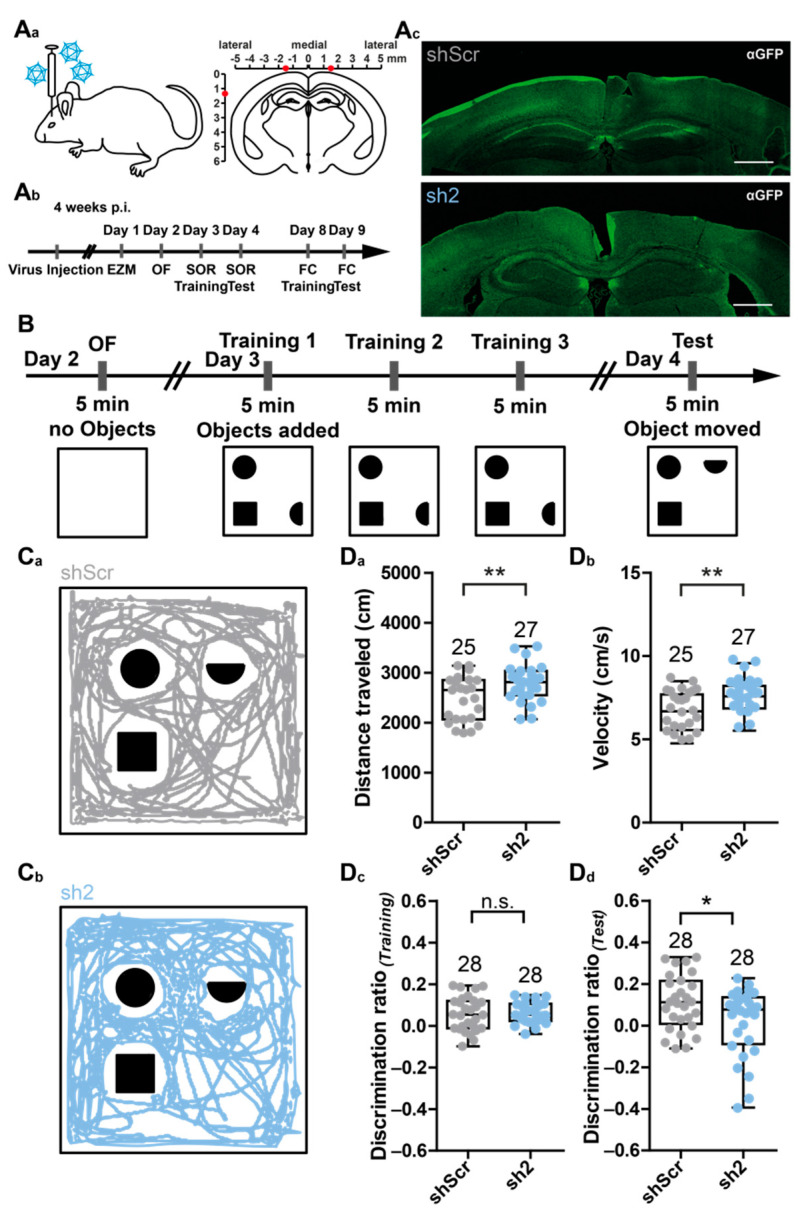
Stereotaxic injection of rAAVs into the dorsal hippocampus and effects of HCN2 loss on spatial object recognition (SOR). (**A**) Injection of rAAVs into hippocampi of mice. (**A_a_**) Schematic showing bilateral injections of rAAV9 virions coding for shScr or sh2 into the dorsal hippocampi of mice. (**A_b_**) Timeline of stereotaxic injections and behavioral experiments (EZM, elevated zero maze test; OF, open field test; SOR, spatial object recognition test; FC, fear conditioning test). (**A_c_**) Representative immunofluorescent images showing expression of the eGFP-reporter (green) in rAAV9-shScr (control, upper panel) or rAAV9-sh2 (HCN2 knock-down, lower panel) bilaterally injected hippocampi. Scale bar 500 µm. (**B**) Schematic showing the timeline and arena settings of the SOR test. (**C**) Tracks of mice bilaterally injected with (**C_a_**) rAAV9-shScr (gray) or (**C_b_**) rAAV9-sh2 (blue) are shown for the testing session. (**D**) Analysis of the behavior of injected mice during SOR testing. Analysis included (**D_a_**) distance traveled during testing session, (**D_b_**) velocity of movement during testing session, (**D_c_**) discrimination ratio between displaced and non-displaced objects during training session, and (**D_d_**) discrimination ratio between displaced and non-displaced objects during testing session. Data are depicted as boxplots. Numbers above boxplots indicate the number of tested animals. Statistical significance was assessed using the unpaired two-tailed Student’s *t*-test, * *p* < 0.05; ** *p* < 0.01.

To investigate potential consequences of a loss of HCN2 on animal behavior, 8-week-old mice were injected with virions encoding shScr or sh2. The spatially restricted delivery of viral suspensions was achieved by bilateral stereotaxic injections targeting the dorsal part of the hippocampus (Figure 3A) and monitored by examining eGFP reporter gene expression in coronal sections (Figure 3B). Behavioral experiments were chosen to cover some of the most important functions of the hippocampal formation in controlling murine behavior (Figure 3C). Thus, the spatial object recognition (SOR, Figure 3) test was used to assess changes in hippocampus-related spatial memory and discrimination abilities. The SOR is based on the spontaneous tendency of mice to spend more time exploring an object that has been relocated compared to already known, non-displaced objects [25].

Mice injected with sh2-encoding virions showed an increase in overall locomotor activity compared to mice injected with shScr-encoding virions, resulting in an increase in the distance traveled (shScr: 2483 ± 444.4 cm; sh2: 2791 ± 369.8 cm) and velocity of movement (shScr: 6.9 ± 1.2 cm/s; sh2: 7.8 ± 1.0 cm/s) (Figure 3C_a_,C_b_). Notably, both groups showed no preference for any of the objects during the training session, resulting in a rather low discrimination ratio (shScr: 0.058 ± 0.083; sh2: 0.063 ± 0.051) (Figure 3C_c_). However, shScr-treated mice showed an increased discrimination ratio during the testing session (shScr: 0.11 ± 0.13), whereas the sh2-treated mice were even worse in discriminating the objects compared to the training session (sh2: 0.02 ± 0.16) (Figure 3C_d_). This result suggested that sh2-treated mice had difficulties to recognize the displaced object.

Taken together, the results of the behavioral experiments indicated that the injection of sh2-encoding virions neither influenced anxiety-related behavior (Figure S3), nor fear-related memory retrieval (Figure S4). However, it caused deficiencies in spatial object memory and discrimination abilities (Figure 3) and induced a robust increase in locomotor activity in the elevated zero maze test (Figure S5).

### 2.3. Molecular and Immunological Analyses of HCN2 Knock-Down

**Figure 4:**
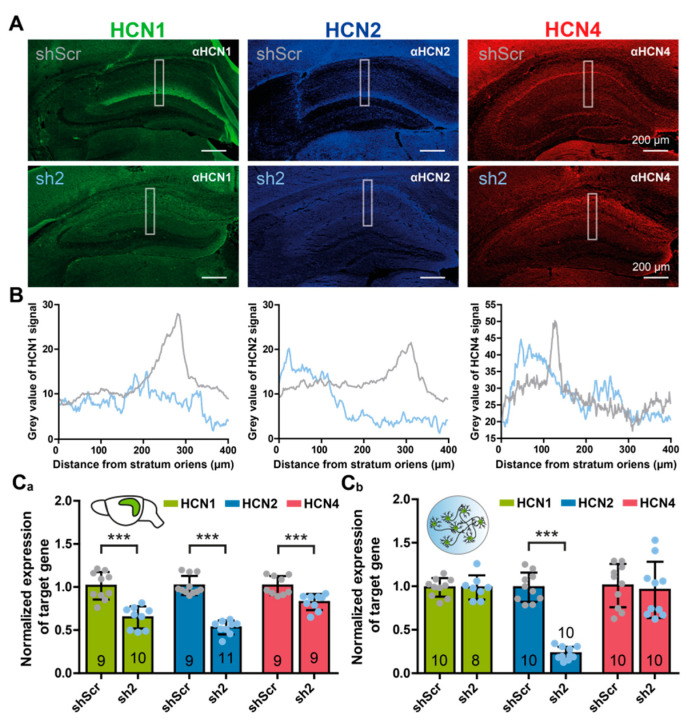
HCN isoform expression changes after loss of HCN2. (**A**) Representative immunofluorescent images showing expression of HCN-channel isoforms 1 (green), 2 (blue), and 4 (red) in rAAV9-shScr- (upper panel) or rAAV9-sh2 (lower panel)-injected hippocampi. Isoforms were stained using subunit-specific antibodies combined with fluorescently labeled secondary antibodies. White rectangles indicate region for (**B**) quantification of immunofluorescence intensities of HCN1-, HCN2-, and HCN4-specific staining. Intensities were measured from the stratum oriens of the cornu amonis 1 (CA1) region to the dorsal part of the dentate gyrus (DG) granule cell layer. (**C**) Quantification of transcript levels by qPCR. (**C_a_**) Quantitative PCR analysis of *hcn1*, *hcn2*, and *hcn4* transcript levels of rAAV-injected brains, and (**C_b_**) rAAV-treated PHNs. Expression levels were calculated and normalized to shScr-treated controls. Data were obtained from indicated numbers of injected hippocampi, or PHN cultures obtained from at least 3 different animals. Results are depicted as mean ± SD. Statistical significance was assessed using the unpaired two-tailed Student’s *t*-test, *** *p* < 0.001.

To visualize the localization of HCN subunits 1, 2, and 4 in the dorsal hippocampus, we performed immunohistochemical staining of tissue sections from animals injected with shScr- or sh2-encoding virions (Figure 4). Both expression of HCN1 and HCN2 channel proteins in shScr-injected animals were restricted to the cornu ammonis (CA) subfield 1 (Figure 4A). Consistent with previous reports [14,16], expression of HCN1 and HCN2 was organized in a gradient of increasing density along the dendrites of CA1 pyramidal neurons, reaching a maximum in the stratum lacunosum-moleculare (slm) (Figure 4A,B). Expression of HCN4 in shScr-injected animals was detected in the granule cell layer of the dentate gyrus (DG) and also in the stratum pyramidale of the CA subfields (Figure 4A). Injection of sh2-encoding virions dramatically changed the expression patterns of each of the HCN channel subtypes. Both HCN1 and HCN2 expression were not organized in a gradient-like pattern 5 weeks post-injection, and HCN4 expression was no longer restricted to the pyramidal cell layer of the CA1 subfield (Figure 4).

To validate the HCN2 knock-down on the transcript level qRT-PCR, we performed analyses of the hippocampi of shScr- and sh2-treated animals (Figure 4C_a_). Notably, in sh2-treated animals, the *hcn2* transcript level was reduced to 53.2 ± 8.0% compared to shScr-treated controls. The sh2-treatment also reduced *hcn1* and *hcn4* transcript levels to 64.8 ± 12.8% and 82.6 ± 9.3% of shScr-treated controls, respectively. To verify if the reduction of *hcn1* and *hcn4* transcripts observed in sh2-treated animals was due to unspecific binding of the sh2 shRNA to *hcn1* or *hcn4* mRNA, we re-evaluated a potential cross-reactivity of sh2-RNA in PHNs (Figure 4C_b_). The sh2-encoding construct reduced *hcn2* transcript levels in PHNs to 22.8 ± 7.4% compared to shScr-treated control neurons. In contrast to the results obtained from the hippocampi of rAAV-injected animals, the sh2-RNA did not cause a reduction in *hcn1* or *hcn4* mRNA levels (*hcn1*: 98.5 ± 13.9% and *hcn4:* 95.8 ± 32.3%) in PHNs (Figure 4C_b_). These results ruled out the possibility that the reduction in transcript expression levels of the *hcn1* and *hcn4* genes in the hippocampus was evoked by unspecific binding of the sh2-RNA to mRNAs coding for the orthologous proteins.

**Figure 5:**
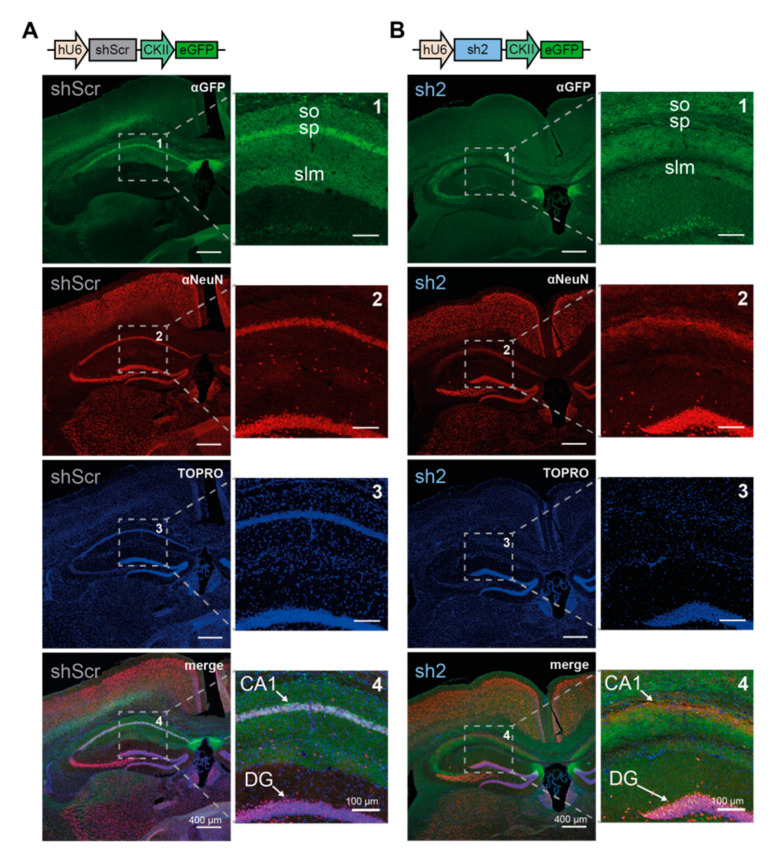
Loss of hippocampal CA1 pyramidal cell layer 5 weeks post-injection of rAAV9-sh2 and control virions. Representative immunofluorescent images showing expression of the eGFP-reporter (green), the neuronal marker protein NeuN (red), and the nuclear marker TOPRO (blue) in (**A**) rAAV9-shScr or (**B**) rAAV9-sh2 bilaterally injected dorsal hippocampi. Animals were sacrificed 5 weeks post-injection. Proteins were stained using specific primary antibodies combined with fluorescently labeled secondary antibodies. Enlargements and arrows show CA1 pyramidal cell layer and the dorsal part of the DG granule cell layer of the hippocampus. Cartoons of the rAAV-delivered constructs are displayed above the immunofluorescent images. so: stratum oriens; sp: stratum pyramidale; slm: stratum lacunosum-moleculare.

Additional immunohistochemical staining of the fluorescent marker eGFP (co-expressed with the shRNA-encoding constructs), the neuronal marker NeuN, and the nuclear marker TOPRO were performed to visualize changes in the overall architecture of the dorsal hippocampal formation (Figure 5). While shScr-injected hippocampi showed a distinct fluorescent signal of eGFP in the CA1 subfield, sh2-injected hippocampi showed a diffuse expression of eGFP in various regions, including the medial part of the DG. Injection of shScr-encoding virions into the dorsal hippocampus resulted in eGFP signals, especially in the stratum pyramidale (sp), harboring somata of CA1 pyramidal neurons; in the stratum lacunosum-moleculare (slm), harboring dendrites of CA1 pyramidal neurons; and in the stratum oriens (so), harboring local branches of the axons of CA1 pyramidal neurons. In contrast, injection of sh2-encoding virions into the dorsal hippocampus resulted in eGFP signals completely lacking the stratum pyramidale of the CA1 subfield (Figure 5A,B, upper panel). Notably, fluorescent signals of both the neuronal marker NeuN and the nuclear marker TOPRO indicated a loss of neurons in the CA1 subfield of sh2-injected hippocampi, which was not visible in shScr-injected hippocampi. The prominent fluorescent signals showing somata (NeuN) and nuclei (TOPRO) in the stratum oriens were completely absent in mice injected with sh2-encoding virions (Figure 5B, medial panels). Contrasting these dramatic effects in the CA1 region, sh2-encoding virions had no obvious effects on the gross architecture of the DG (Figure 5B).

To unravel if the loss of the CA1 pyramidal cell layer would progress to other regions of the hippocampal formation (e.g., DG), we analyzed fluorescent images from animals sacrificed 9 weeks post-injection of shScr- or sh2-encoding virions (Figure S6). Similar to the effects observed 5 weeks post-injection, fluorescent signals of soma (NeuN) and nuclei (TOPRO) were absent in mice 9 weeks after injection of sh2-encoding virions (Figure S6A,B). The cell-loss occurring in the CA1 region, however, did not seem to expand to the granule cells of the DG.

**Figure 6:**
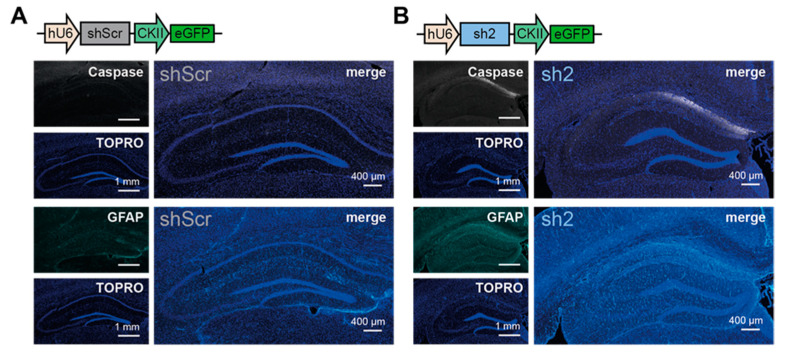
Apoptotic marker expression in dorsal hippocampal CA1 region 5 weeks post-injection of rAAV9-sh2 and control virions. Representative immunofluorescent images showing expression of the apoptosis marker active caspase-3 (Caspase, gray) and astrogliosis-enriched glial fibrillary acidic protein (GFAP, cyan). Animals were injected with (**A**) rAAV9-shScr virions or (**B**) rAAV9-sh2 virions. Animals were sacrificed 5 weeks post-injection. Proteins were stained using specific antibodies combined with fluorescently labeled secondary antibodies. Nuclei were stained with TOPRO (blue). Cartoons of the rAAV-delivered constructs are displayed above the images.

To gain further insight into the mechanism accompanying the loss of the CA1 pyramidal cell layer in sh2-treated mice, we used active (cleaved) caspase-3 (Caspase) and glial fibrillary acidic protein (GFAP) as indicators for the adverse tissue responses [26] (Figure 6). At 5 weeks post-injection, fluorescent signals of active caspase-3, a main component of apoptosis in eukaryotic cells [27], was very high, especially in the CA1 subfield of sh2-injected hippocampi compared to shScr-injected hippocampi (Figure 6, upper panel). Notably, at 9 weeks post-injection, caspase-3 signals of sh2-injected hippocampi were almost indistinguishable from those of shScr-treated control hippocampi (Figure S7, upper panel), suggesting a decline or final stage of the apoptotic mechanisms after 9 weeks. Moreover, at 5 weeks post-injection, also GFAP signals, serving as a marker for glial scars after injury, increased in the CA1 subfield of sh2-injected hippocampi compared to controls (Figure 6, lower panel). The GFAP signals did not decline in sh2-injected animals 9 weeks post-injection (Figure S7, lower panel). Moreover, at 9 weeks post-injection, shScr-treated control animals showed similar elevated levels of GFAP expression in the CA1 subfield and in the DG, indicating that GFAP expression either generally might increase with aging, independent of the molecular identity of the injected virus, or might be caused by the stereotaxic surgery procedure.

In summary, the results of the molecular and immunological analyses of in vivo HCN2 knock-down indicated that injection of sh2-encoding virions caused a severe but specific apoptotic degeneration of the CA1 pyramidal cell layer. Injection of shScr-encoding control virions, however, neither resulted in changes of gene expression levels, marker protein localization, nor overall hippocampal architecture.

## 3. Discussion

The goal of this study was to characterize the consequences of HCN2 knock-down in hippocampal neurons in vitro and in vivo. The mammalian genome encodes four HCN channel subunits (HCN1–4) [28] that are known to control electrical properties of neurons, for example by determining and stabilizing the resting membrane potential [1]. In addition, HCN channels play crucial roles in generating rhythmic cellular activity and thereby participate in cardiac pacemaking [2], as well as in modulating the sleep and wake cycle in the thalamocortical system [3].

### 3.1. In Vitro HCN2 Knock-Down in PHNs

The knock-down of the HCN2 subunit in PHNs neither changed the resting membrane potential, I_h_-current activation kinetics, nor I_h_-current activation potentials (Figure 2), as previously reported for a HCN2 knock-out mouse model in CA1 hippocampal neurons [29]. At a first glance, these observations were astonishing, since *hcn2* transcript levels in the hippocampus were relatively high and accounted for approximately 70% of all *hcn* transcripts in these neurons [21]. However, HCN2 homomeric channels were reported to have a higher single channel conductance compared to homomeric HCN1 or HCN4 channels [30]. In agreement with these properties, the knock-down of HCN2 in PHNs led to a strong reduction in I_h_-current amplitudes and densities (Figure 2). Furthermore, the activation kinetics and potentials of native I_h_-currents recorded in wildtype or shScr-transduced neurons were similar to the kinetics and activation potentials of homomeric HCN2-channel currents recorded in transgenic HEK293 cell lines [21]. Altogether, these observations suggest that HCN2 subunits are important contributors to heteromeric HCN channels and thus of the I_h_-current in PHNs. Additionally, the formation of HCN2/HCN1 heteromers [31,32], HCN2/HCN4 heteromers [33], or even HCN1/HCN4 heteromers [34] might equip hippocampal neurons with a powerful molecular repertoire of HCN channels to generate a variety of different I_h_-currents with distinct electrophysiological and biochemical characteristics encoded by a relatively small number of genes [35]. The existence of several channel β-subunits, scaffolding proteins, and regulatory proteins, i.e., TRIP8b, caveolin-3, or MiRP1, increase the variability of HCN channels and may further expand the functional properties of these proteins in vivo [10].

For ion channels becoming activated at sub-threshold membrane potentials, the remarkable plasticity regarding HCN channel properties raised the question for which processes this plasticity can be utilized. A prominent feature, especially of HCN1 channels, is their conductance at the resting membrane potential [36]. The presence of HCN channels induces a permanent depolarization of the resting membrane potential, due to the inward current conducted by these non-inactivating channels [37]. Thereby, HCN channels can counteract both hyperpolarizing and depolarizing input by either producing a depolarizing inward current due to I_h_-current activation, or by inducing membrane hyperpolarization due to I_h_-current deactivation [38]. Rather than solely stabilizing the resting membrane potential, HCN channels are thus perfectly suited to fine-tune a neuron’s response to depolarizing or hyperpolarizing external stimuli [39]. These integrating properties were intensively studied in CA1 hippocampal neurons [14]. Integration of EPSPs at the dendrites must be precisely controlled, both temporally and spatially, to generate appropriate output(s) at the soma. This is accomplished by organizing HCN channels in a gradient-like pattern of increasing density from the soma to the distal end of the dendrites in CA1 pyramidal neurons (Figure 4A,B). This spatial distribution facilitates EPSP time courses that become increasingly shortened with the distance from the soma [19]. A loss of, e.g., HCN1, leads to increased postsynaptic responses in CA1 neurons, which triggers induction of perforant path long-term potentiation (LTP) and thereby enhances hippocampal-dependent learning and memory processes. These findings emphasize a role of HCN channels in dendritic integration and thus behavior [40]. In contrast to the HCN1 knock-out, a HCN2 knock-out did not constrain LTP in the perforant path [41]. In accordance with this finding, time courses of EPSCs were not decelerated in PHNs treated with sh2-encoding virions causing HCN2 subunit knock-down. However, decay time constants of miniature EPSCs and spontaneous EPSCs were increased compared to control conditions (Figure 1 and Figure S1). Even though the electrophysiological recordings of sh2-treated PHNs did not show an increase in I_h_-current kinetics, there might be a shift from heteromeric HCN1/HCN2 channels to HCN1-dominated homomeric channels, as described for I_h_-currents in HCN2 knock-out mice [29,41]. This would lead to an increase in resting HCN1 channel conductance and to an acceleration of EPSPs. These observations might underpin the role of HCN1 and HCN2 heteromeric channels in balancing excitation/inhibition in neurons. Still, the precise role of HCN2 channels to regulate presynaptic neurotransmission and dendritic integration in vivo remains elusive.

### 3.2. HCN2 Knock-Down in the Dorsal Hippocampus of Young Adult Mice

To investigate the role of the HCN2 isoform in vivo, we injected rAAVs encoding sh2-RNA or shScr-RNA into the dorsal hippocampus of 8-week-old mice. Previous studies have demonstrated that mice with reduced I_h_-currents due to the complete loss of HCN1, HCN2, or the auxiliary scaffolding protein TRIP8b showed antidepressant-like behavior [29,40,41,42], along with subunit-specific behavioral changes such as impaired motor-learning or improved short- and long-term spatial learning and memory [43]. The behavioral changes observed in our study upon injection of sh2-encoding virions were not in accordance with previous reports. Instead, the injected animals showed no changes in anxiety- or fear-related behaviors. However, the injected animals showed increased locomotor activity and had deficits in spatial memory. Although a clear reduction of *hcn2* transcripts was detected in sh2-injected mice (approximately 53%), unexpected reductions in the transcript levels of HCN1- and HCN4-encoding genes were uncovered (Figure 4C_a_). These “unspecific” knock-down effects were not detected in PHNs transduced with the same sh2-encoding virions (Figure 4C_b_), supporting the notion that the reduction of *hcn1* and *hcn4* transcript levels was not due to off-target effects of the HCN2-targeting shRNA. The immunohistochemical analysis finally shed some light on these rather surprising results. The staining with the neuronal marker NeuN showed that the observed behavioral changes could be explained by neurodegenerative processes, ultimately leading to a loss of the hippocampal CA1 pyramidal cell layer (Figure 5 and Figure 6). The loss of these neurons was accompanied by increased levels of active caspase-3 and GFAP expression compared to control conditions (Figure 6), indicating an apoptotic mechanism [44]. This apoptotic effect is unlikely to originate from experimental issues, as previous in silico and in vitro experiments using the same virus batch were inconspicuous and the application of the shScr-encoding control virions ruled out the possibility of cytotoxic effects emerging from the hU6 promoter that has been reported previously [26].

Interestingly, despite the finding that eGFP expression originating from the viral constructs was found in the dentate gyrus (DG), there were no indications for apoptotic mechanisms in the DG. Notably, DG neurons express only basal levels of HCN channel isoforms compared to the CA1 region (Figure 4A). Even after 9 weeks post-injection, there were no indications for further spreading of the neurodegenerative and apoptotic processes (Figures S6 and S7). Thus, the degeneration of neurons in the CA1 region might be attributed to the knock-down of the HCN2 subunit during week 8–12 of the animal’s life span. Supporting this idea, CA1 pyramidal neurons of the dorsal hippocampus (DHC) express more HCN2 subunits compared to CA1 pyramidal neurons of the ventral hippocampus (VHC) [45]. This indicates that the physiological function of HCN2 might be more relevant to the DHC than to the VHC. In accordance with this finding, the changes in spatial memory observed after injection of rAAV9-sh2 are mainly attributed to DHC-related defects [46], while changes in anxiety- or fear-related behaviors are mainly attributed to VHC [47,48].

Moreover, HCN channel expression is not only tightly regulated in space, but also in time [8,39,49]. This emphasizes that interfering with the exquisitely regulated protein expression and distribution could lead to dramatic changes in neuronal homeostasis. Even though there are no reports on compensatory up- or downregulations of gene expression in HCN1 or HCN2 knock-out animals, the interference with gene expression at a certain time point during postnatal stages might have severe functional implications [50].

Mechanistically, a knock-down of HCN2 might lead to a disturbance of the excitation–inhibition balance. HCN channels are regulated by neuronal activity [51]. The magnitude of somatic I_h_-currents is dependent on excitatory synaptic activity, which has been proposed as a homeostatic mechanism for regulating neuronal excitability [52]. This mechanism may have an additional homeostatic role by narrowing the time window for coincidence detection during increased neuronal activity [53]. Vice versa, the coincidence detection window would broaden with decreasing I_h_-current activity. Interfering with this mechanism might cause over-excitation and ultimately could lead to neurodegeneration due to the cytotoxic actions of excessive glutamate receptor activity [54]. Supporting this idea, propofol, a commonly used anesthetic acting on both GABA_A_ receptors and HCN channels at clinically relevant concentrations [55], induces apoptosis of CA1 pyramidal neurons in mice [56]. Whether the neurodegenerative effect of propofol is caused by its direct actions on HCN channels has to be experimentally proven. Additionally, point mutations in the *hcn2* gene were uncovered in patients suffering from febrile seizures [57] or epilepsy [58,59]. These mutations were thought to lower the threshold of action potentials and thereby strongly increase excitability. However, although the specificity and efficacy of the HCN2 knock-down approach has been successfully demonstrated in PHNs, additional and alternative experimental strategies are required to examine the precise temporal and functional implications of HCN2 in CA1 pyramidal cells and on mouse behavior in forthcoming studies.

## 4. Materials and Methods

### 4.1. Primary Hippocampal Neuron Culture

Preparation and cultivation of primary hippocampal neurons (PHNs) was performed as described previously [21]. Briefly, hippocampi from 1–3-day-old mice (C57BL/6 strain from an in-house animal facility) were dissected in ice-cold Hanks’ balanced salt solution (HBSS; Gibco/Thermo Fisher Scientific; Darmstadt, Germany). Hippocampi were incubated in papain solution (DMEM (Gibco/Thermo Fisher Scientific), 25 U·mL^−1^ papain, 1.6 mM L-cysteine, 1 mM CaCl_2_, 0.5 mM EDTA) at 37 °C for 20 min and subsequently transferred to inactivating solution (2.5% (*w*/*v*) trypsin inhibitor, 2.5% (*w*/*v*) albumin in FCS solution consisting of DMEM, 100 U·mL^−1^ penicillin, 100 μg·mL^−1^ streptomycin, and 10% (*v*/*v*) FCS, all from Gibco/Thermo Fisher Scientific), and 0.1% (*v*/*v*) MITO+ serum extender (Corning/Thermo Fisher Scientific) at 37 °C for 5 min. After trituration in FCS solution, cells were counted and plated on coverslips in 4-well plates (Ibidi, Martinsried, Germany) pre-coated with poly-D-lysine (0.2 mg mL^−1^ poly-d-lysine, 50 mM H_3_BO_3_, 25 mM Na_2_B_4_O_7_, pH 8.5) at a density of 300 cells mm^−2^. Cells were maintained in 500 µL NBA medium (Neurobasal A Medium (Gibco/Thermo Fisher Scientific), 100 U·mL^−1^ penicillin, 100 μg·mL^−1^ streptomycin, 2% (*v*/*v*) B27-supplement (Invitrogen/Thermo Fisher Scientific, Darmstadt, Germany), and 1% (*v*/*v*) Glutamax (Gibco/Thermo Fisher Scientific)) at 37 °C, 5% CO_2_, and 95% relative humidity for 14 days. Medium was partially exchanged every 2–3 days. For transduction, recombinant Adeno-associated viral suspensions (rAAVs) were added with a multiplicity of infection of 2 × 10^4^ per neuron, 2–3 days after plating (days in vitro, d.i.v.).

### 4.2. Immunocytochemistry

PHNs cultured on coverslips were rinsed with PBS and fixed in PFA (4% (*w*/*v*) paraformaldehyde in PBS) for 10 min at room temperature (RT). After several rinses with PBS, unspecific binding sites were blocked for 1 h at RT in blocking solution (CT: 5% (*v*/*v*) ChemiBLOCKER (Merck, Darmstadt, Germany), 0.5% (*v*/*v*) Triton X-100). Subsequently, coverslips were incubated with primary antibodies (Table 1) in CT over night at 4 °C or for 4 h at RT, rinsed for several times with PBS and incubated with secondary antibodies (Table 2) in CT for 1 h at RT. Finally, coverslips were washed with PBS before mounting with Aqua-Poly/Mount (Polysciences, Eppelheim, Germany) on microscopy slides. Fluorescent images were obtained with an inverted confocal microscope (TCS SP5II; Leica, Wetzlar, Germany).

### 4.3. Preparation of Tissue

For tissue preparation, newborn animals were cooled on ice and decapitated. Adult animals were anesthetized with 5% isoflurane and decapitated. Fur, muscle, and the lower jaw were removed. The cranium was opened along the main fissure using scissors and forceps, and the brain was removed. For RNA isolation, the hippocampi were isolated in ice-cold Hanks’ balanced salt solution (HBSS; Gibco/Thermo Fisher Scientific) and stored at −80 °C.

### 4.4. Immunohistochemistry

For immersion fixation, whole brains were fixed in 4% (*w*/*v*) PA overnight. For cryo-protection, tissue was incubated in 10% (*w*/*v*) sucrose (in PBS) for 1 h at RT and subsequently in 30% (*w*/*v*) sucrose (in PBS) for 2 days at 4 °C. For cryo-sectioning, tissue was embedded in freezing medium (Tissue Tek, Sakura Finetek, Zouterwoude, the Netherlands) and frozen at −20 °C. Tissue was cut in 18–22 µm thick sections at −22 °C using a cryostat (Microm HM550, Thermo Fisher Scientific). Slices were transferred onto microscope slides (SuperForst Plus, Menzel), air-dried, and stored at −20 °C until further use. For immunological staining, sections were thawed, dried at RT, and encircled with a hydrophobic marker (ImmEdge™ Pen, Vector Laboratories, CA, USA). Staining was performed in a damp chamber. After several rinses with PBS, unspecific binding sites were blocked for 1 h at RT in CT blocking solution. Subsequently, samples were incubated with primary antibodies (Table 3) in blocking solution (CT) and 0.75% (*v*/*v*) Triton X-100 at 4 °C overnight, rinsed for several times with PBS, and then incubated with secondary antibodies (Table 4) in CT and 0.75% (*v*/*v*) Triton X-100 at RT for 1 h. Finally, samples were washed with PBS before embedding samples with Aqua-Poly/Mount (Polysciences, Eppelheim, Germany) under coverslips. Fluorescent images were obtained with an inverted confocal laser scanning microscope (TCS SP5II; Leica, Wetzlar, Germany).

### 4.5. Whole-Cell Patch-Clamp Recordings

Whole-cell patch-clamp recordings were performed at RT following the methods described by Hamill et al. [60]. Patch pipettes with tip resistances between 2.5 and 4 MΩ were fashioned from borosilicate glass with an inner diameter of 0.86 mm and an outer diameter of 1.5 mm (Harvard Apparatus, Holliston, MA, USA) using a temperature-controlled pipette puller (P1000, Sutter Instrument, Novato, CA, USA). Pipettes were filled with intracellular saline solution containing 10 mM KCl, 10 mM NaCl, 120 mM KGluconate, 10 mM EGTA, 10 mM HEPES, 4 mM MgATP, and 0.3 mM NaGTP, adjusted to pH 7.3 with KOH and an osmolality of ≈310 mOsm L^−1^. During the experiments, PHNs were superfused constantly with extracellular saline solution containing 150 mM NaCl, 4 mM KCl, 2 mM CaCl_2_, 2 mM MgCl_2_, and 10 mM HEPES, adjusted to pH 7.4 with NaOH and adjusted to 330 mOsm L^−1^ with glucose. To isolate HCN-mediated I_h_-currents, we blocked glutamate receptor (AMPA/kainate)-mediated currents by 10 µM CNQX (Tocris Bioscience, Ellisville, MI, USA), blocked glutamate receptor (NMDA)-mediated currents by 50 µM D-APV (Tocris Bioscience), blocked GABA_A_ receptor-mediated currents by 25 µM bicuculline (Tocris Bioscience), blocked inwardly rectifying potassium currents by 0.5 mM BaCl_2_ (Sigma-Aldrich, Schnelldorf, Germany), blocked voltage-dependent potassium channels by 3 mM 4-AP (Tocris Bioscience), and blocked voltage-dependent sodium channels by 2 µM TTX (Tocris Bioscience). To isolate miniature EPSCs, we blocked voltage-dependent sodium channels by 2 µM TTX, and blocked GABA_A_ receptor-mediated currents by 25 µM bicuculline. To isolate spontaneous and evoked EPSCs, we blocked GABA_A_ receptor-mediated currents by 25 µM bicuculline. To determine mEPSC properties with reasonable fidelity and to prevent detection of “false events” (due to random noise fluctuations), we analyzed only the mEPSCs with peak amplitudes of >15 pA and a charge criterion of >25 fC [61] using a commercial software (Mini Analysis, Synaptosoft, Version 6.0.3). Whole-cell voltage-clamp and current-clamp recordings were performed using an EPC10 patch-clamp amplifier (HEKA-Elektronik, Lambrecht, Germany) that was controlled by the program Patch Master (version 2.5; HEKA-Elektronik). Electrophysiological data were sampled at 20 kHz and lowpass filtered at 2.9 kHz with a four-pole Bessel-filter. Offset potentials, electrode capacity, and membrane capacity were compensated manually. PHNs were voltage-clamped at −70 mV. The liquid junction potential between intracellular and extracellular solutions was calculated and also compensated by adjusting the offset potential. Series resistance was compensated between 60 and 80% with a time constant of 100 μs.

### 4.6. Production and Purification of Recombinant Adeno-Associated Viruses (rAAVs)

To knock-down *hcn2* transcript levels by RNAi, we cloned shRNA fragments (control (shScr) F: CAACAAGATGAAGAGCACCAA, R: TTGGTGCTCTTCATCTTGTTG; hcn2-specific (sh2) F: CCATGCTGACAAAGCTCAAAT, R: TTTGAGCTTTGTCAGCATGG) into pENN-CaMKIIeGFP vector provided by the University of Pennsylvania Vector Core (Philadelphia, PA, USA) containing the human U6 (hU6) promoter 5′ upstream to the shRNA-encoding fragment. For calcium imaging in PHNs, the eGFP reporter gene was replaced by a GCaMP6f-WPRE encoding cassette, isolated from plasmid #100834 (Addgene; Watertown, MA, USA), which was a gift from James M. Wilson. The construct was called pENN.AAV.CamKII.GCaMP6f.WPRE.SV40. Recombinant Adeno-associated viral (rAAV) particles were prepared by triple-transfection of HEK293 cells (ATCC; #CRL-1573) using a modified calcium phosphate coprecipitation method [62]. HEK293 cells were cultivated in DH10 medium (DMEM + GlutamaxTM, 10% (*v*/*v*) FBS, 1% (*v*/*v*) antibiotics/antimycotics (all from Gibco/Thermo Fisher Scientific)) at 37 °C, 5% CO_2_, and 95% relative humidity. Transfections were performed with a vector encoding the transgenic viral genome flanked by AAV2 (wt) inverted terminal repeats (ITRs) and the helper plasmids pXX6-80 and pRC2/9 (R.J. Samulski, University of Florida, Gainesville, USA) providing the proteins for DNA replication and capsid assembly of rAAVs, respectively. Twenty-four hours after transfection, the medium was exchanged for hunger medium (DMEM + GlutamaxTM, 2% (*v*/*v*) FBS, and 1% (*v*/*v*) antibiotics/antimycotics (all from Gibco/Thermo Fisher Scientific)). Seventy-two hours after transfection, cells were harvested in PBS-M/K (130 mM NaCl, 2.5 mM KCl, 1 mM MgCl_2_, 70 mM Na_2_HPO_4_, 30 mM NaH_2_PO_4_, pH 7.4) and centrifuged (200× *g*, 4 °C, 5 min). Cell pellets were re-suspended in lysis buffer (150 mM NaCl, 50 mM Tris/HCl, pH 8.5), and cells were lyzed by five freeze/thaw cycles. Free nucleic acids were digested with benzonase (50 U·mL^−1^; Merck Millipore, Darmstadt, Germany) for 30 min at 37 °C. After centrifugation (5000× *g*, 4 °C, 30 min), the rAAV suspension was sub-layered with iodixanol gradient solutions (10%, 20%, 40%, 60%) and centrifuged (rotor Ti 70; 264,000× *g*, 4 °C, 2 h). Viral particles were collected in the 40% iodixanol phase, sterile filtered (0.2 µm pore size), and further purified using Amicon Ultra Centrifugal Filters (Ultracel-100k, 15 mL; Merck Millipore). For determination of genomic titers, viral genomes were isolated using the DNeasy Blood and Tissue Kit (Qiagen, Hilden, Germany) according to the supplier’s protocol, and quantitative PCR was performed using a primer pair framing an identical segment of the eGFP- or GCaMP6f-encoding constructs (see Table 5).

### 4.7. Stereotaxic Injections of rAAV Vectors

Mice (4-week-old male mice, Mus musculus, C57BL/6J (Charles River, MA, USA)) were kept in groups of 4 animals in greenline cages (Tecniplast, Hohenpeißenberg, Germany) under an inverted 12:12 light/dark cycle at 21 ± 2 °C, 50–70% relative humidity, food and water ad libitum, nesting material available, and habituated for 4 weeks before they underwent stereotaxic injection of rAAV suspensions. For injection of viral suspensions, a stereotaxic setup from World Precision Instruments, Inc. (Sarasota, FL, USA) was used. The setup included a Stereotaxic Frame with 45° zygomatic bars for fixation of the cranium on two axes, an UltraMicroPump III (UMP3) with SYS-Micro4 MicroSyringe pump controller for microinjection, and a NanoFil 10 μL syringe with a 33G beveled replacement NanoFil needle for minimal intrusion. Animals were deeply anesthetized with 2.5% isoflurane prior to and during surgery. Additionally, animals received analgetic treatment with an intraperitoneal injection of Novalgin (200 mg·kg^−1^) and local injection of Bupivacain (80 µL, 2.5 mg·mL^−1^). During surgery, an animal temperature controller with a heating plate and a temperature probe (World Precision Instruments, Inc.) was used to keep body temperature constant. The fur at the surgical site was removed, skin was cleaned, and finally the site was prepared with Kodan Tinktur forte (Schülke, Hamburg, Germany). An incision was made to expose the top of the cranium. Bilateral holes were drilled into the cranium according to the injection coordinates with a micro driller (Ideal Micro Drill, Stoelting, Wood Dale, IL, USA). Injections were performed bilaterally in the CA1 region of the dorsal hippocampus at stereotaxic coordinates −1.9 mm anteroposterior (AP) relative to the bregma, ±1.5 mm mediolateral (ML), and −1.4 mm dorsoventral (DV). Mice were randomly assigned to receive suspensions of rAAV9 (pENN-hU6-shScr-CaMKII-eGFP; control) or rAAV9 (pENN-hU6-sh2-CaMKII-eGFP; *hcn2*-specific) and experimenters were blinded for which suspensions they injected. Animals received 1 μL viral suspension (2.5 × 10^9^ viral particles in total) per hippocampus with a rate of 0.2 μL min^−1^. After injection, the incision was sutured with Ethilon Monofil (Ethicon, Somerville, NJ, USA). After suturing the fur, the skin was cleaned and treated with Octenisept (Schülke). At 4 h, 24 h, and 48 h post-injection, mice received analgetic treatment with intraperitoneal injections of carprofen (5 mg·kg^−1^) and were scored according to the experimental procedures approved by the LANUV. One week before starting behavioral experiments, mice were single housed and control (shScr injected) or knock-down (sh2 injected) mice were handled for 2 min per day for 3 consecutive days before the first behavioral experiments were performed.

### 4.8. Spatial Object Recognition

Behavioral testing and tissue collection was performed during the morning of the light phase. Data collection and analysis of behavioral experiments were performed automatically using the ANY-maze (Stoelting, Wood Dale, IL, USA) video tracking system. For spatial object recognition, control (shScr injected) or knock-down (sh2 injected) mice were exposed to a custom-made, rectangular open field arena (30.5 cm × 38.5 cm). An internal visual cue was placed on one of the four arena walls and three distinct objects, i.e., a glass bottle, a rectangular metal column, and a half round shaped plastic cylinder were placed in the arena at specified locations. During three training sessions on the same day, mice were allowed to freely explore the environment and objects for 6 min in each session. Mice were placed back to their home cages for 3 min between the training sessions. After 24 h, mice were placed back in the arena for the testing phase. The same three objects were present in the arena, but one of the three objects (the half round shaped plastic cylinder) was displaced to a novel spatial position. Mice were allowed to explore the environment and the objects for 6 min. The third training sessions and the testing sessions were scored for exploratory behavior (animal’s snout was within approximately 1 cm distance of an object). Discrimination between the objects was calculated using a discrimination ratio (DR), defined as the absolute difference in the time spent exploring the novel and familiar objects divided by the total time spent exploring the objects, which takes into account individual differences in the total amount of exploration [63]. Testing sessions were also scored for the total distance moved and velocity of movement.

### 4.9. Quantification of Gene Expression by Real-Time PCR

Total RNA from rAAV-transduced PHNs was isolated after 14–15 d.i.v. using the RNeasy Mini Kit (Qiagen) according to the supplier´s protocol. Total RNA from whole hippocampi was isolated by grinding the frozen tissue to a powder using a Teflon bar, followed by adding 200 µL of RLT+ Buffer (Qiagen) to the frozen powder. Further homogenization was achieved by passing the lysate 5–10 times through a 25-gauge needle attached to a 1 mL syringe until a homogeneous lysate was achieved. To minimize RNA loss, we washed the syringe with 150 µL of RLT+ Buffer (Qiagen). First-strand cDNA synthesis was performed using Oligo-dT primers (Qiagen) and Moloney Murine Leukemia Virus reverse transcriptase (M-MLV-RT, Life Technologies/Thermo Fisher Scientific) according to the supplier’s protocol. Briefly, cDNA synthesis was performed in a final volume of 25 µL. A total of 1 µg of RNA was mixed with 0.5 µg Oligo-dT Primers and denatured at 65 °C for 10 min. Samples were transferred quickly to 4 °C for 2 min for hybridization of Oligo-dT primers to mRNA. Synthesis of cDNA was performed in First-Strand Buffer (1×), 1 mM dNTPs, 40 U RNaseOUT™ (Invitrogen/Thermo Fisher Scientific), 10 mM DTT, and 400 U M-MLV-RT at 37 °C for 1 h. The enzyme was inactivated at 65 °C for 10 min, and cDNA samples were aliquoted and stored at −80 °C until use. Thermocycling was performed in a LightCycler 1.5 (Roche, Mannheim, Germany) using the QuantiTect SYBR Green PCR Kit (Qiagen). qPCR reactions were performed on 2 µL aliquots of first-strand cDNA samples in a total volume of 20 µL. Gene-specific primers (Table 5) were designed in silico and synthesized by Eurofins (Ebersberg, Germany). Specificity of primers was confirmed via BLAST analysis. For normalization, *gapdh* was used as a reference gene. The *gapdh* primers were designed to bind in exons separated by an intron of 134 bp to check for genomic impurities as described previously [21]. For calibration, three standard fragment probes covering fragment numbers of at least five orders of magnitude were amplified (in duplicates). qRT-PCR reactions on cDNA samples were also performed in duplicate. Data were analyzed using the delta-delta Ct-method [64].

**Table 5:** Sequences of qPCR primer pairs. Primer sequences, accession numbers, melting temperatures, and amplicon sizes for target and reference genes used in qPCR experiments.

Target Gene	Sequence	T_m_ (°C)	Fragment Length (bp)
eGFP/GCaMP6f JQ064510.1/MW139900.1	F: GACGTAAACGGCCACAAGTTC R: GAAGTCGTGCTGCTTCATGTG	60	189
mGAPDH NM_008084.2	F: GGCATTGTGGAAGGGCTCATG R: GCCCACAGCCTTGGCAGC	62	150
mHCN1 NM_010408.3	F: CTCAGTCTCTTGCGGTTATTACG R: TGGCGAGGTCATAGGTCATG	62	91
mHCN2 NM_008226.2	F: ATCGCATAGGCAAGAAGAACTC R: CAATCTCCTGGATGATGGCATT	60	102
mHCN4 NM_001081192.1	F: GCATGATGCTTCTGCTGTGT R: GCTTCCCCCAGGAGTTATTC	60	123

### 4.10. Data Analysis

Electrophysiological data were analyzed using FitMaster (version 2; HEKA-Elektronik) or Igor Pro (version 6; Wavemetrics, Lake Oswego, OR, USA). Data are represented as mean ± SD (standard deviation) or as box and whisker plots. The two-tailed unpaired Student’s *t*-test was applied for the calculation of *p*-values using GraphPad Prism (version 5; GraphPad Software Inc., La Jolla, CA, USA). A *p*-value of < 0.05 was considered significant.

## Figures and Tables

**Table 1:** Primary antibodies used for immunocytochemistry. List of primary antibodies applied for immunocytochemistry. Supplier: Chemicon, Merck/Sigma-Aldrich, Darmstadt, Germany; Synaptic Systems, Goettingen, Germany; Invitrogen/Thermo Fisher Scientific, Darmstadt, Germany. Abbreviations: ch, chicken; gp, guinea pig; rb, rabbit.

Antigen	Source	Dilution	Supplier
GFP	ch	1:1000	Chemicon (ab16901)
HCN1	gp	1:500	in house
HCN2	rb	1:500	in house
HCN4	rb	1:500	in house
MAP2	rb	1:1000	Synaptic Systems (188 002)
TOPRO-3		1:1000	Invitrogen (T3605)

**Table 2:** Secondary antibodies used for immunocytochemistry. List of secondary antibodies applied for immunocytochemistry. Supplier: Dianova, Hamburg, Germany. Abbreviations: ch, chicken; dk, donkey; gp, guinea pig; rb, rabbit.

Antibody	Source	Dilution	Supplier
α ch Cy2	dk	1:200	Dianova (703-225-155)
α gb Cy3	dk	1:500	Dianova (706-165-148)
α rb Cy3	dk	1:500	Dianova (711-165-152)
α rb Dy488	dk	1:500	Dianova (711-485-152)

**Table 3:** Primary antibodies used for immunohistochemistry of tissue sections. List of primary antibodies applied for immunohistochemistry. Supplier: Abcam, Berlin, Germany; Merck/Sigma-Aldrich, Darmstadt, Germany; Chemicon, Merck/Sigma-Aldrich, Darmstadt, Germany; Invitrogen/Thermo Fisher Scientific, Darmstadt, Germany. Abbreviations: ch, chicken; ms, mouse; rb, rabbit; rt, rat.

Antigen	Source	Dilution	Supplier
Cleaved caspase-3	rb	1:50	Abcam (ab2302)
GFAP	ms	1:500	Merck/Sigma-Aldrich (G3893)
GFP	ch	1:1000	Chemicon (ab16901)
HCN1 7C3	rt	1:5	in house
HCN2 3G7	rt	1:10	in house
HCN4 PG2-1A4	rt	1:2	in house
NeuN	rb	1:500	Abcam (ab104225)
TOPRO-3		1:1000	Invitrogen (T3605)

**Table 4:** Secondary antibodies used for immunohistochemistry of tissue sections. List of secondary antibodies applied for immunohistochemistry. Supplier: Dianova, Hamburg, Germany. Abbreviations: ch, chicken; dk, donkey; ms, mouse; rb, rabbit; rt, rat.

Antigen	Source	Dilution	Supplier
α ch Cy2	dk	1:200	Dianova (703-225-155)
α ms Cy3	dk	1:400	Dianova (715-165-150)
α rb Cy3	dk	1:400	Dianova (711-165-152)
α rt Cy3	dk	1:400	Dianova (712-165-153)

## Data Availability

Data and materials are available from the corresponding author upon request.

## Supplementary Materials

Supplementary Materials can be found at https://www.mdpi.com/article/10.3390/ijms22136699/s1.

## Abbreviations

AAV	Adeno-associated virus
EPSP	excitatory postsynaptic potential
EZM	elevated zero maze test
FC	fear conditioning test
FCS	fetal calf serum
GFP	green fluorescent protein
HCN	hyperpolarization-activated and cyclic nucleotide-gated ion channel
HEK293	human embryonic kidney cells
OF	open field test
PHN	primary hippocampal neuron
qRT-PCR	quantitative real-time polymerase chain reaction
RNAi	inhibitory RNA
shRNA	short hairpin RNA
SOR	spatial object recognition test
TTX	tetrodotoxin
