# Supplementary material for: Loss of HCN2 in Dorsal Hippocampus of Young Adult Mice Induces Specific Apoptosis of the CA1 Pyramidal Neuron Layer
Source: Int J Mol Sci. 2021 Jun 22;22(13):6699. doi: 10.3390/ijms22136699 (PMC8269412; doi:10.3390/ijms22136699)
Supplement: Supplementary file 1 [file ijms-22-06699-s001.zip › ijms-1240697-supplementary.pdf]

Article

Loss of HCN2 in dorsal hippocampus of young adult mice induces specific apoptosis of the CA1 pyramidal neuron layer

Matthias Deutsch, Carina Stegmayr , Sabine Balfanz and Arnd Baumann

Supplementary Material

Methods

Action potential and sEPSC recordings

For a detailed description of the patch-clamp recordings see main Material and Methods section. To isolate action potentials in primary hippocampal neurons (PHNs), glutamate (AMPA/kainite) receptor-mediated currents were blocked by 10 µM CNQX, glutamate (NMDA) receptor-mediated currents were blocked by 50 µM D-APV, and $GABA_A$ receptor-mediated currents were blocked by 25 µM Bicuculline. To isolate spontaneous excitatory post synaptic currents (sEPSCs), $GABA_A$ receptor-mediated currents were blocked by 25 µM Bicuculline. To determine sEPSC properties with reasonable fidelity and to prevent detection of "false events" (due to random noise fluctuations), only sEPSCs with peak amplitudes of >15 pA and a charge criterion of >25 fC (1) were analyzed using a commercial software (Mini Analysis, Synaptosoft, Version 6.0.3).

Calcium-imaging

To monitor intracellular Ca^{2+}-fluctuations in PHNs, Ca^{2+}-responses in rAAV9 (pENN-hU6-shRNA-CaMKII-GCaMP6f-WPRE) transduced neurons were triggered by voltage step stimulations of varying intensities. Coverslips with PHNs were placed in a custom-made recording chamber surrounded by platinum wires (diameter of 0.5 mm) attached to an external stimulation unit (NIHON Electronic Stimulator 1001, NIHON KOHDEN, Rosbach, Germany). Cells were superfused constantly with extracellular saline solution containing 150 mM NaCl, 4 mM KCl, 2 mM $MgCl_2$, 2 mM $CaCl_2$, 10 mM HEPES, pH 7.4. Live cell imaging was performed using an Olympus BX50WI microscope body (Olympus, Tokio, Japan) and an ANDOR-TM camera (iXONEM+ DU-897D-CS0BV, Andor Solis, Oxford Instruments, Abingdon, UK) for signal detection. For excitation, a 470 nm LED (THORLABS, M00462613) was used. The light was guided through a dichroic mirror reflecting wavelengths ≤ 498 nm onto the sample. The excitation light was focused on the sample via the objective and the emitted light was guided through the dichroic mirror passing wavelengths ≥ 498 nm. Photons were collected by the camera. The system was operated with an ANDOR SOLIS software (Andor Solis X-2747). During measurements, exposure time and acquisition rate was automatically adjusted. The time units were recalculated to seconds based on the number of frames taken per second.

Behavioral Experiments

Behavioral testing and tissue collection was performed during the morning of the light phase. Data collection and analysis of behavioral experiments were performed automatically using the ANY-maze (Stoelting, Wood Dale, IL, USA) video tracking system.

Elevated zero maze test

For elevated zero maze testing, control (shScr injected) or HCN2 knock-down (sh2 injected) mice were exposed to a custom made zero maze with a height of 70 cm, a boardwalk width of 5 cm and an outside circumference of 200 cm. It consisted of two open quadrants and two closed quadrants. Each mouse was allowed to investigate the apparatus for 5 min. Sessions were scored for the time spent in open and closed quadrants, distance moved, velocity of movement, as well as the number of transitions between quadrants. Transitions were scored when the center point of the body left the former quadrant. Time spent in the open quadrants vs. the closed quadrants was calculated.

Open field test

For open field testing, control (shScr injected) or HCN2 knock-down (sh2 injected) mice were exposed to a custom-made open field arena consisting of a rectangular open field (30.5 cm x 38.5 cm) for 6 min. Quadrants were defined as central (inner area more than 5 cm distant from apparatus wall) and peripheral (within 5 cm distance of apparatus wall) zones. Sessions were scored for the time spent in the central and peripheral zones, the number of transitions between central and peripheral zones, as well as distance moved, and velocity of movement. Transitions were scored when the center point of the body left the former zone. Time spent in the central zone vs. time spent in the peripheral zone was calculated.

Contextual Fear conditioning

Contextual fear conditioning was performed using the Ugo Basile fear conditioning system (Stoelting). Animals received three training sessions in enclosed rectangular conditioning chambers. Control (shScr injected) or HCN2 knock-down (sh2 injected) animals were exposed to the conditioning context for 148 s, followed by a 2 s 0.75 mA footshock. Animals were removed from the chambers 30 s after receiving a footshock and were placed back to their home cage for 3 minutes between the training sessions. After 24 h, animals were tested for memory retention by returning them to the conditioning chamber for a single 5 min context test. Training and testing sessions were recorded and freezing behavior as well as distance moved, and velocity of movement was measured using automated scoring software.

Table S1: Electrophysiological properties of hippocampal neurons. Electrophysiological parameters were measured using whole-cell voltage clamp and current clamp recordings. For a detailed description see main Materials and Methods section. Parameters are depicted as mean ± standard deviation. Numbers of cells tested are shown in parentheses.

**Fig. 1**	**E_m**	**R_{in}**	**I_h** (-130 mV)	**Sag** (-130 mV)	**I_h $V_{1/2}$**	**I_h τ** (-130 mV)	**Sag half-width** (-130 mV)
	**(mV)**	**(MΩ)**	**(pA)**	**(mV)**	**(mV)**	**(s)**	**(s)**
shScr	**-68.33** ± 4.82 **(24)**	**481.0** ± 153.9 **(23)**	**120.3** ± 76.1 **(17)**	**12.09** ± 2.85 **(12)**	**-108.4** ± 3.2 **(9)**	**0.427** ± 0.182 **(24)**	**0.143** ± 0.039 **(13)**
sh2	**-68.45** ± 3.42 **(22)**	**458.9** ± 127.7 **(19)**	**67.8** ± 37.8 **(18)**	**7.77** ± 2.04 **(12)**	**-108.5** ± 5.71 **(13)**	**0.412** ± 0.211 **(24)**	**0.132** ± 0.042 **(15)**

**Fig.2**	**mEPSC** amplitude	**mEPSC** frequency	**mEPSC** decay time	**mEPSC** charge
	**(pA)**	**(Hz)**	**(ms)**	**(fC)**
shScr	**-13.84** ± 1.35 **(15)**	**1.47** ± 0.95 **(17)**	**10.68** ± 0.87 **(14)**	**-72.28** ± 10.76 **(15)**
sh2	**-13.06** ± 1.35 **(17)**	**1.28** ± 0.74 **(22)**	**9.37** ± 1.35 **(16)**	**-58.53** ± 11.0 **(17)**

**Fig.S1**	**AP** threshold	**AP** amplitude	**AP** half-width	**AHP** amplitude
	**(mV)**	**(mV)**	**(ms)**	**(mV)**
shScr	**-49.27** ± 2.34 **(11)**	**78.77** ± 10.04 **(11)**	**2.38** ± 0.31 **(11)**	**7.27** ± 1.82 **(10)**
sh2	**-50.57** ± 3.92 **(12)**	**79.42** ± 17.54 **(12)**	**2.37** ± 0.62 **(12)**	**8.02** ± 2.79 **(10)**

**Fig. S2**	**sEPSC** amplitude	**sEPSC** frequency	**sEPSC** decay time	**sEPSC** charge
	**(pA)**	**(Hz)**	**(ms)**	**(fC)**
shScr	**-26.16** ± 3.25 **(15)**	**1.29** ± 0.66 **(12)**	**9.56** ± 1.8 **(20)**	**-119.0** ± 13.78 **(14)**
sh2	**-23.42** ± 3.96 **(12)**	**1.0** ± 0.59 **(12)**	**7.81** ± 1.32 **(13)**	**-85.47** ± 21.74 **(10)**

Supplementary Figures

Figure S1

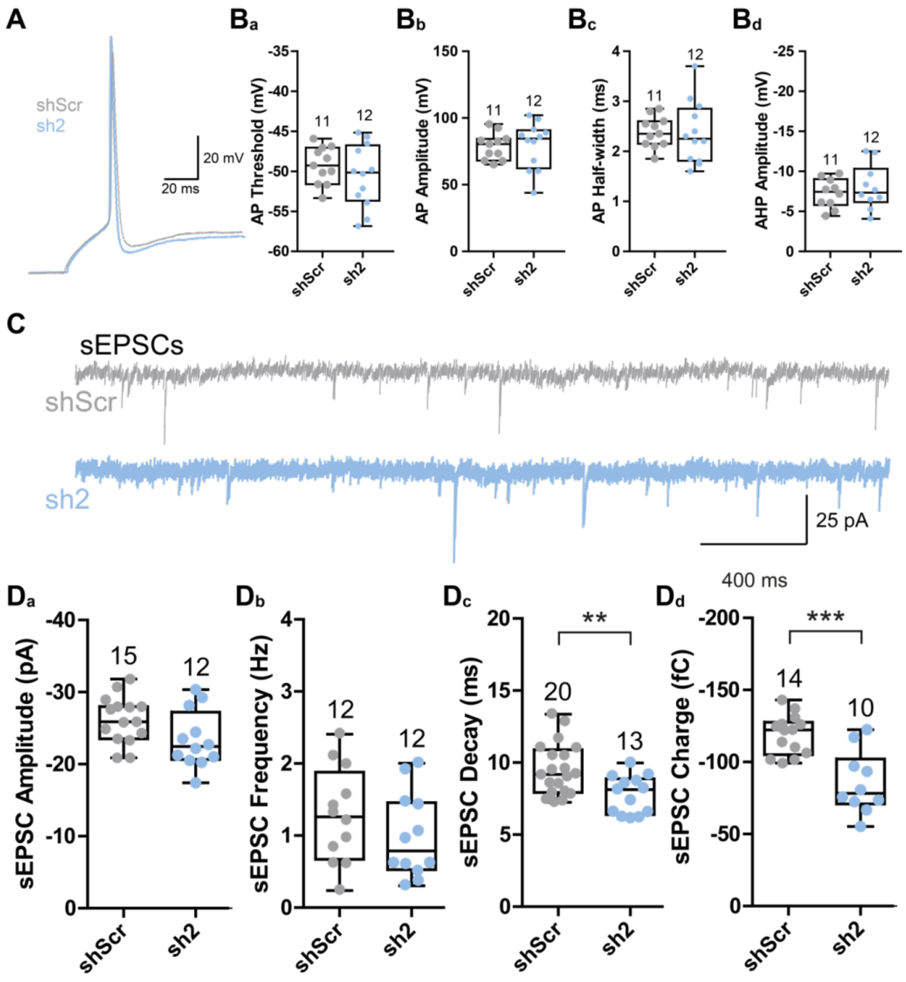

Figure S1. Effects of HCN2 knock-down on action potential properties and sEPSCs of primary hippocampal neurons. (A) Representative voltage traces showing initial action potentials (APs) of whole-cell patch-clamp recordings derived from PHNs treated with shScr (control) or sh2 (HCN2 knock-down) encoding rAAV9. The initial APs were evoked by injecting depolarizing currents in 10 pA increments until threshold was reached. **(B)** Characteristic AP parameters. (B_a) Influence of HCN2 knock-down on AP threshold, (B_b) AP amplitude, (B_c) AP half-width, and (B_d) amplitude of after-hyperpolarization (AHP) in PHNs. **(C)** Representative current traces showing sEPSCs in PHNs treated with shScr (control) or sh2 (HCN2 channel knock-down) encoding rAAV9. **(D)** Influence of HCN2 channel knock-down on (D_a) sEPSC amplitude, (D_b) sEPSC frequency, (D_c) sEPSC decay time, calculated by fitting the decay phase by a mono-exponential decay equation, and (D_d) on sEPSC charge, calculated by the integral of the synaptic events. Data are depicted as boxplots. Data were obtained from indicated numbers of neurons from at least 3 independent neuronal cultures. Statistical significance was assessed using the unpaired two-tailed Student´s t test, **$p<0.01$, ***$p<0.001$.

Figure S2

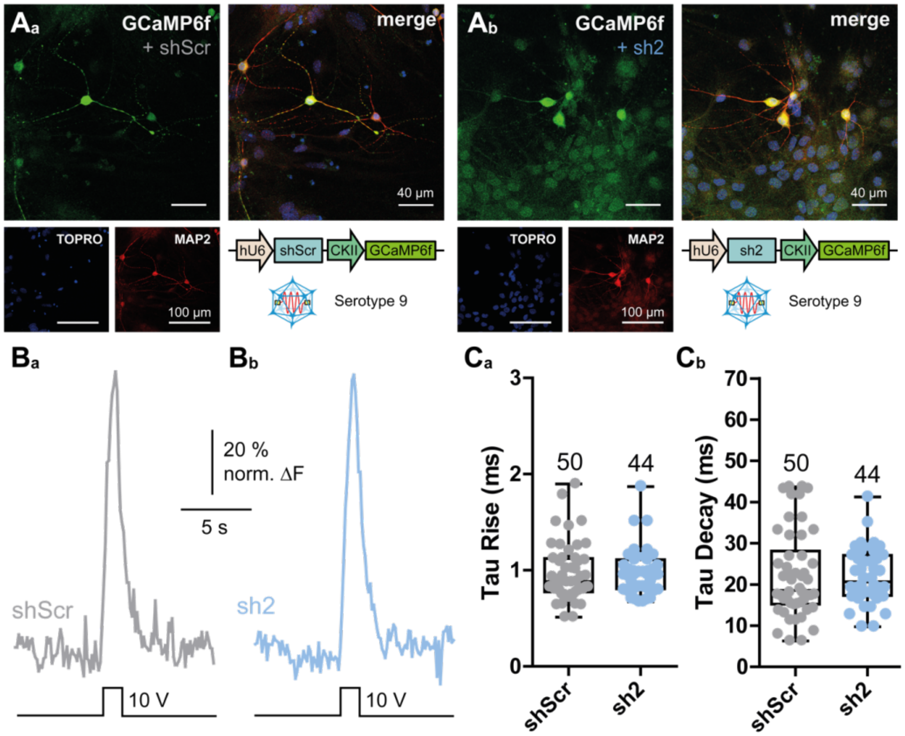

Figure S2. Effects of HCN2 knock-down on intracellular Ca^{2+} responses of PHNs. (A) Representative immunofluorescent images of PHNs transduced with rAAV9 encoding GCaMP6f-shScr (A_a) or GCaMP6f-sh2 **(**A_b). GCaMP6f was labeled using an anti-GFP antibody and neurons were stained using a microtubule-associated protein 2 (MAP2) antibody. Primary antibodies were combined with fluorescently labeled secondary antibodies (GFP, green; MAP2, red). Nuclei were labeled with TOPRO (blue). Cartoons of the rAAV-delivered constructs are displayed below the merged immunofluorescent images. **(B)** Representative fluorescent responses and stimulation protocol of GCaMP6f in PHNs either transduced with (B_a) GCaMP6f-shScr or (B_b) GCaMP6f-sh2. **(C)** The rising and decay phase of the fluorescence responses to a stimulus intensity of 10 V for 200 ms were fitted by an exponential growth equation to determine the (C_a) rise time. The decay phase was fitted by a one phase decay equation to determine the (C_b) decay time. Results are depicted as boxplots. Data were obtained from indicated numbers of neurons from at least 3 independent neuronal cultures. Statistical significance was assessed using the unpaired two-tailed Student´s t test.

Figure S3

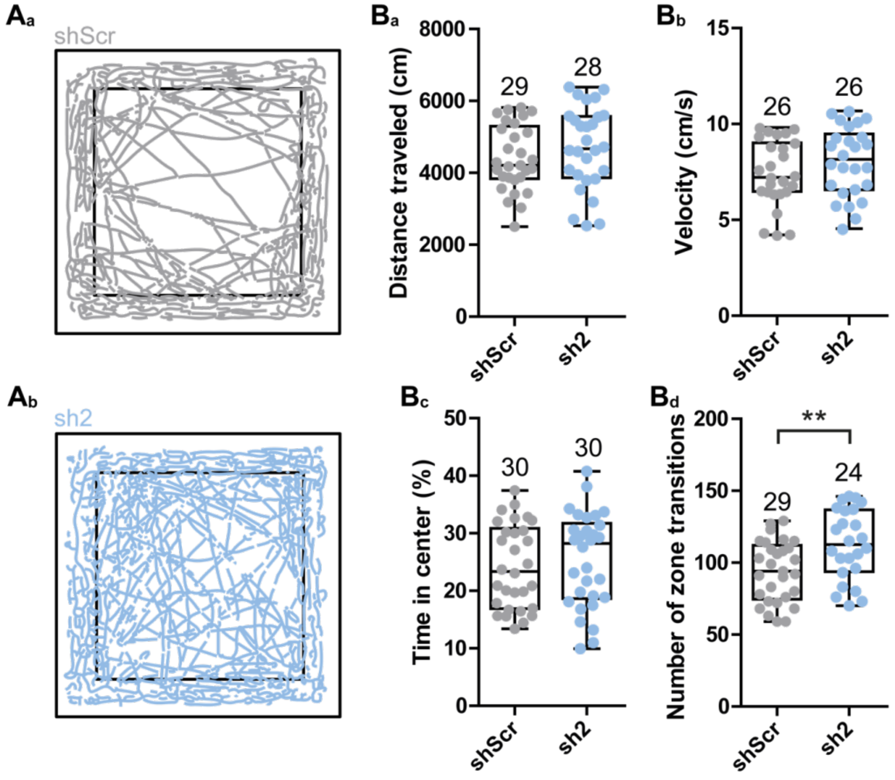

Figure S3. Influence of rAAV injection into dorsal hippocampus on open field (OF) behavior. (A) Schematic showing the open field (OF) arena and tracks of mice bilaterally injected with (A_a) rAAV9-shScr (gray) or (A_b) rAAV9-sh2 (blue). For analysis, the arena is separated into a peripheral and a central area (inner square). **(B)** Behavior of injected animals was scored for (B_a) distance traveled, (B_b) velocity of movement, (B_c) time spent in the central area of the arena, and (B_d) number of transitions between peripheral and central areas of the arena. Data were obtained from indicated numbers of injected animals. Statistical significance was assessed using the unpaired two-tailed Student´s t test, $^{**}p<0.01$.

Figure S4

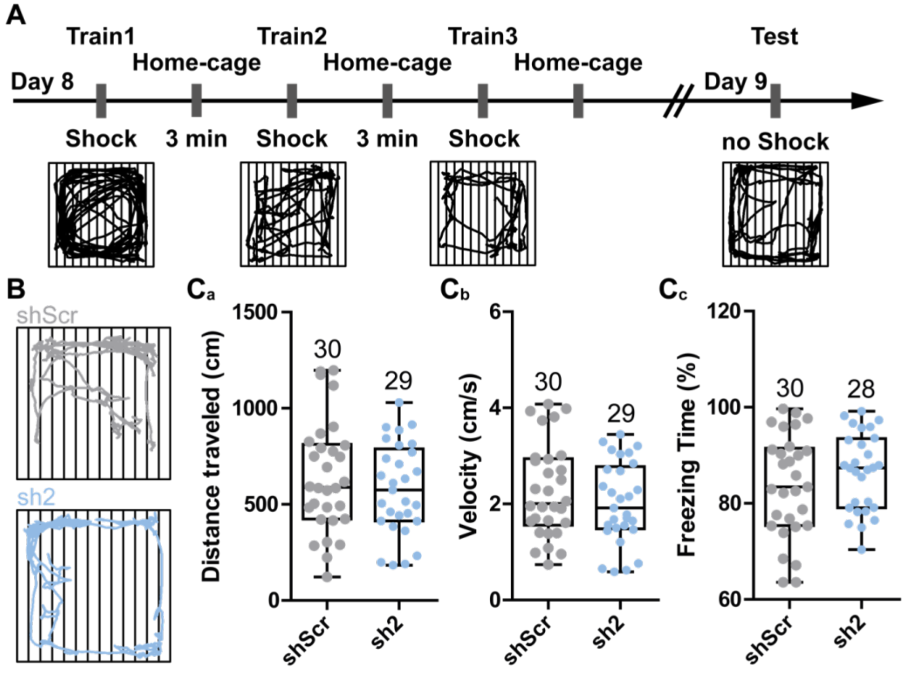

Figure S4. Influence of rAAV injection into dorsal hippocampus on fear conditioning (FC). (A) Schematic showing the timeline and arena for the fear conditioning (FC) test. **(B)** Tracks of mice bilaterally injected with rAAV9-shScr (gray) or rAAV9-sh2 (blue) are shown. **(C)** In the testing sessions behavior was scored for (C_a) distance traveled, (C_b) velocity of movement, and (C_c) freezing time. Data are depicted as boxplots. Data were obtained from indicated numbers of injected animals. Statistical significance was assessed using the unpaired two-tailed Student´s t test.

Figure S5

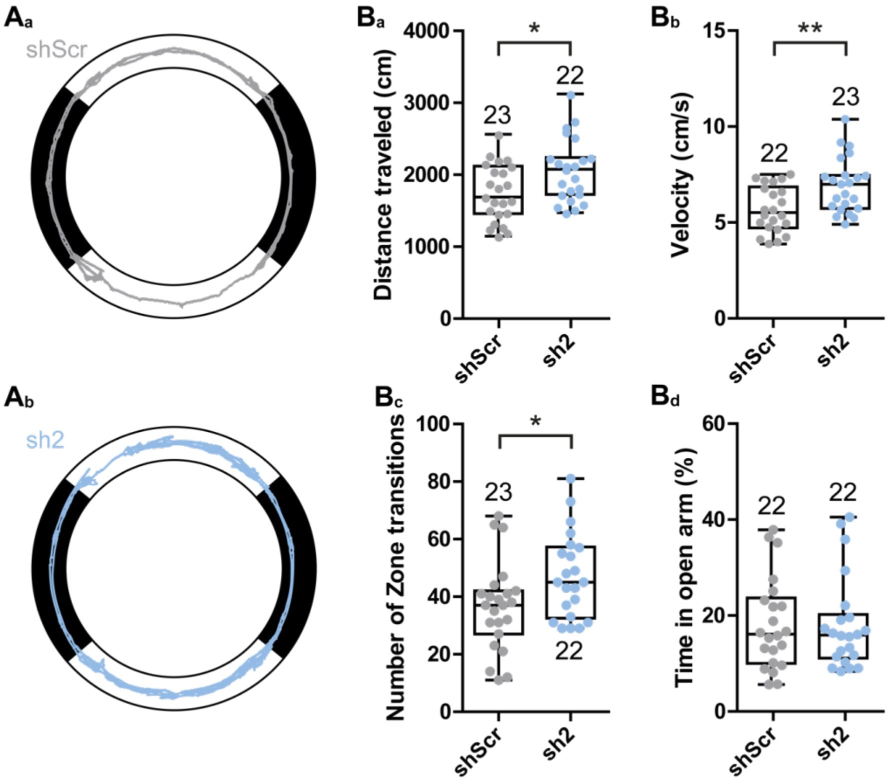

Figure S5. Influence of rAAV injection into dorsal hippocampus on elevated zero maze test (EZM). (A) Schematic showing the elevated zero maze (EZM) arena and tracks of mice bilaterally injected with (A_a) rAAV9-shScr (gray) or (A_b) rAAV9-sh2 (blue). The white areas represent the open quadrants, while the black areas represent the closed quadrants of the maze. **(B)** Behavior was scored for (B_a) distance traveled, (B_b) velocity of movement, (B_c) number of transitions between open quadrants and closed quadrants of the maze, and (B_d) time spent in the open arm of the maze. Data are depicted as boxplots. Data were obtained from indicated numbers of injected animals. Statistical significance was assessed using the unpaired two-tailed Student´s t test, *$p<0.05$; **$p<0.01$.

Figure S6

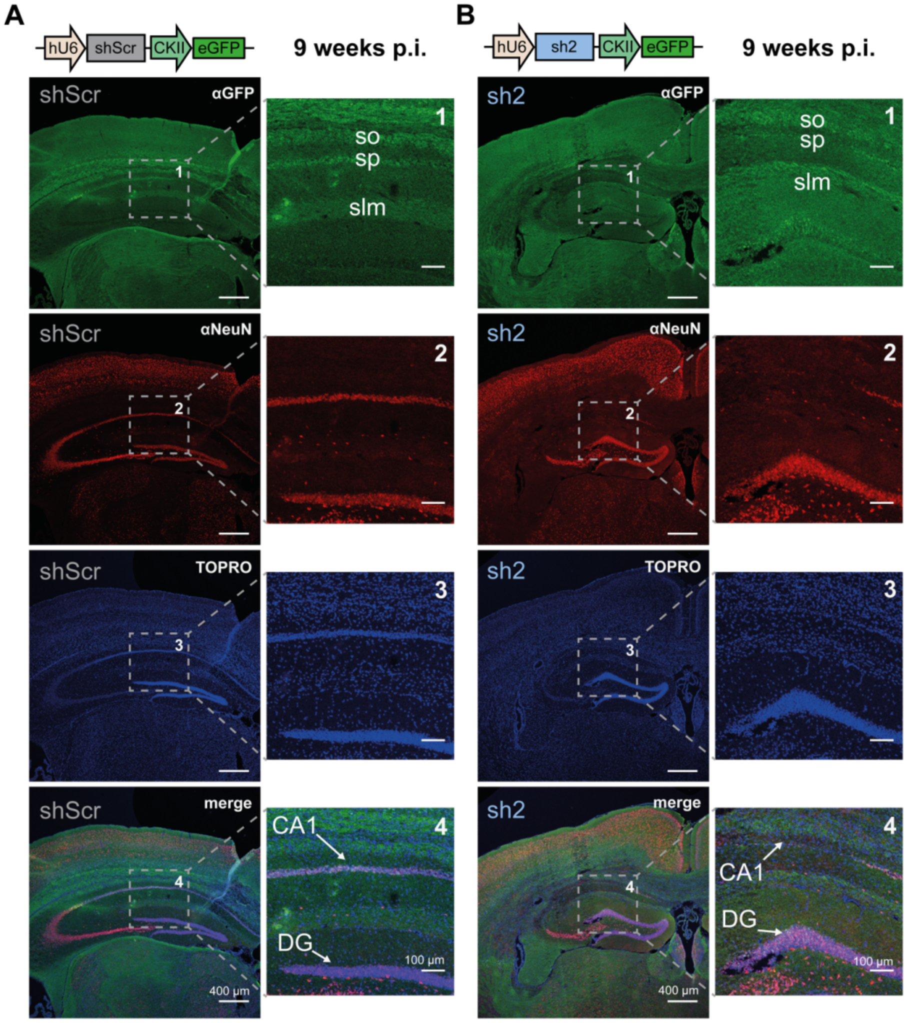

Figure S6. Loss of hippocampal CA1 pyramidal cell layer 9 weeks post injection. Representative immunofluorescent images showing expression of the fluorescent reporter eGFP (green), the neuronal marker protein NeuN (red), and the nuclear marker TOPRO (blue) in **(A)** rAAV9-shScr or **(B)** rAAV9-sh2 bilaterally injected hippocampi. Animals were sacrificed 9 weeks post injection. Proteins were stained using specific primary antibodies combined with fluorescently labeled secondary antibodies. Enlargements and arrows show the cornu amonis 1 (CA1) pyramidal cell layer and the dorsal part of the dentate gyrus granule (DG) cell layer of the hippocampus. Cartoons of the rAAV-delivered constructs are displayed above the immunofluorescent images. so: stratum oriens; sp: stratum pyramidale; slm: stratum lacunosum-moleculare.

Figure S7

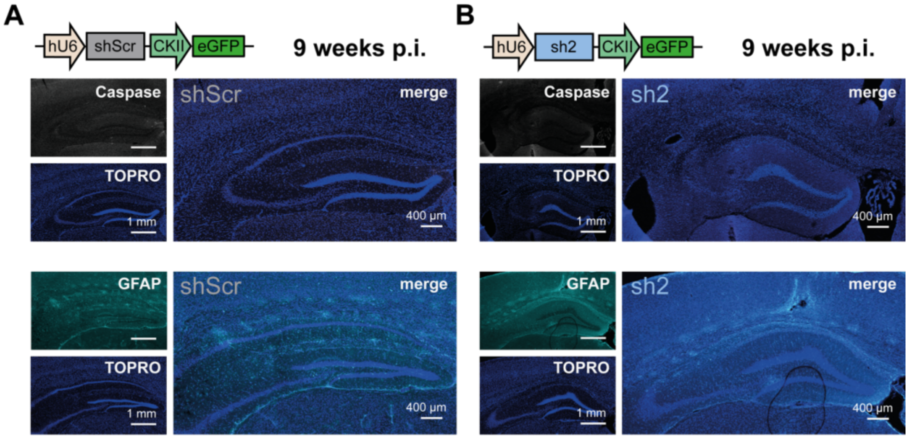

Figure S7. Markers of rAAV9-sh2-induced tissue degeneration in the hippocampal CA1 region. Representative immunofluorescent images showing expression of the apoptosis marker active caspase-3 (Caspase, gray) and astrogliosis enriched glial fibrillary acidic protein (GFAP, cyan). Animals were injected with **(A)** rAAV9-shScr virions or **(B)** rAAV9-sh2 virions. Animals were sacrificed 9 weeks post injection. Proteins were stained using specific antibodies combined with fluorescently labeled secondary antibodies. Nuclei were stained with TOPRO (blue). Cartoons of the rAAV-delivered constructs are displayed above the immunofluorescent images.

References:

1. Guzman, R.E.; Schwarz, Y.N.; Rettig, J.; Bruns, D. SNARE force synchronizes synaptic vesicle fusion and controls the kinetics of quantal synaptic transmission. *J. Neurosci.* **2010**, 30, pp. 10272-10281.
